# Interferon-α2 Auto-antibodies in Convalescent Plasma Therapy for COVID-19

**DOI:** 10.1007/s10875-021-01168-3

**Published:** 2021-11-12

**Authors:** Matthijs P. Raadsen, Arvind Gharbharan, Carlijn C. E. Jordans, Anna Z. Mykytyn, Mart M. Lamers, Petra B. van den Doel, Henrik Endeman, Johannes P. C. van den Akker, Corine H. GeurtsvanKessel, Marion P. G. Koopmans, Casper Rokx, Marco Goeijenbier, Eric C. M. van Gorp, Bart J. A. Rijnders, Bart L. Haagmans

**Affiliations:** 1 Viroscience Department, Erasmus MC, Rotterdam, The Netherlands; 2 Department of Medical Microbiology and Infectious Diseases, Erasmus MC, Rotterdam, The Netherlands; 3 Intensive Care Department, Erasmus MC, Rotterdam, The Netherlands

**Keywords:** COVID-19, SARS-CoV-2, Interferon alpha, Auto-antibodies, Convalescent plasma

## Abstract

**Purpose:**

To study the effect of interferon-α2 auto-antibodies (IFN-α2 Abs) on clinical and virological outcomes in critically ill COVID-19 patients and the risk of IFN-α2 Abs transfer during convalescent plasma treatment.

**Methods:**

Sera from healthy controls, cases of COVID-19, and other respiratory illness were tested for IFN-α2 Abs by ELISA and a pseudo virus–based neutralization assay. The effects of disease severity, sex, and age on the risk of having neutralizing IFN-α2 Abs were determined. Longitudinal analyses were performed to determine association between IFN-α2 Abs and survival and viral load and whether serum IFN-α2 Abs appeared after convalescent plasma transfusion.

**Results:**

IFN-α2 neutralizing sera were found only in COVID-19 patients, with proportions increasing with disease severity and age. In the acute stage of COVID-19, all sera from patients with ELISA-detected IFN-α2 Abs (13/164, 7.9%) neutralized levels of IFN-α2 exceeding physiological concentrations found in human plasma and this was associated with delayed viral clearance. Convalescent plasma donors that were anti-IFN-α2 ELISA positive (3/118, 2.5%) did not neutralize the same levels of IFN-α2. Neutralizing serum IFN-α2 Abs were associated with delayed viral clearance from the respiratory tract.

**Conclusions:**

IFN-α2 Abs were detected by ELISA and neutralization assay in COVID-19 patients, but not in ICU patients with other respiratory illnesses. The presence of neutralizing IFN-α2 Abs in critically ill COVID-19 is associated with delayed viral clearance. IFN-α2 Abs in COVID-19 convalescent plasma donors were not neutralizing in the conditions tested.

**Supplementary Information:**

The online version contains supplementary material available at 10.1007/s10875-021-01168-3.

## Introduction

SARS coronavirus 2 (SARS-CoV-2) causes both upper and lower respiratory tract infections in humans with a broad spectrum of disease severity, ranging from asymptomatic viral replication and shedding to life-threatening COVID-19. The latter is characterized by severe bilateral pneumonia resulting in acute hypoxia, systemic inflammation, and hypercoagulability [1, 2]. Several host factors which predispose an individual to develop severe disease during SARS-CoV-2 infection have been identified. Advanced age, obesity, and associated comorbidities such as heart failure, chronic kidney disease, and diabetes are well known to adversely impact immunity and are risk factors for developing severe infections, including COVID-19 [3]. More specifically, impaired type I IFN activity in COVID-19 patients has been associated with ineffective control of viral replication and excessive inflammation [4]. Genetic defects in Interferon Response Factor 7, Toll-Like Receptor 3, and Interferon Alpha Receptor 1 have been identified as underlying deficient IFN responses in some cases of severe COVID-19 [5]. Auto-antibodies against type I IFNs (IFN-Abs) were found in approximately 10% of patients with life-threatening COVID-19, resulting in an auto-immune phenocopy of inborn errors of type I IFN signaling [6]. These IFN-Abs were not found in mild cases and were rare in healthy individuals (~0.3%). Based on these findings, the authors noted the potential risk posed by convalescent plasma donated by individuals recovering from severe COVID-19 and suggest either excluding these donors or testing them first. Auto-Abs against IFN-α2 were found in 4/116 of convalescent blood plasma donors previously hospitalized with COVID-19, with 2 (1.5 %) neutralizing IFN α2 in a cell-based assay [7].

Randomized controlled trials conducted in patients with severe COVID-19 pneumonia indicate convalescent plasma treatment in hospitalized patients is safe but ineffective [8]. Most COVID-19 patients have already developed virus-neutralizing Abs by the time they are admitted to the hospital, rendering those in the donor plasma redundant [9]. New trials are underway, administering virus-neutralizing convalescent plasma earlier in the course of the disease. This has the potential to aid viral clearance, but may also carry the risk of transferring harmful IFN neutralizing Abs to these patients at a time when they are more reliant on their early innate antiviral response. Monoclonal antibody therapies employ the same mechanism of action, with reduced risk of potentially harmful off-target effects, but with some concerns for selecting viral escape mutants. Interim trial results show early administration of monoclonal antibody therapy in mild COVID-19 patients at high risk of developing severe disease results in a decrease in viral load compared to placebo and a reduction in symptom scores in the week following infusion [10, 11].

In this study, we compared detection rate and neutralizing potential of IFN-α2 Abs in sera from critically ill COVID-19 patients and ICU patients with other infectious respiratory diseases. We determined whether neutralizing IFN-α2 Abs are present in sera of COVID-19 convalescent plasma donors and whether these are transferred to patients following convalescent plasma therapy. Subsequently, we determined whether the presence of neutralizing IFN-α2 Abs affected clinical and virological outcomes in COVID-19 patients.

## Methods

### Human Samples

Patients admitted to the ICU at the Erasmus MC, Rotterdam, the Netherlands, with severe respiratory disease with suspected infectious etiology, including SARS-CoV-2, were included in a biorepository study. Written informed consent was signed by study subjects or their representatives if they were incapacitated due to severe illness. Serum was obtained on the first day of inclusion into the study and stored at −80 °C until further analysis. The study protocol was approved by the institutional review board of Erasmus MC, Rotterdam, the Netherlands (MEC-2017-417 and MEC-2020-0222). Serum samples taken during the CONCOVID clinical trial (NCT04342182) were kindly made available by the trial investigators. Sera from blood plasma donors were taken at a mean of 51 (SD ± 14 days) days after COVID-19 disease onset. The donors mostly had mild disease, with 15 (13%) having been hospitalized. During the first months of the pandemic, only male donors were recruited for convalescent plasma donation for reasons of urgency, since additional HLA and HNA antibody tests are required in female donors before their plasma can be used [12, 13]. This led to an artificial overrepresentation of men in this cohort. Sera from trial subjects were available of 61 hospitalized severe COVID-19 cases, 28 of whom had received convalescent plasma treatment, while the rest received standard of care. Baseline samples were taken on the day of inclusion, which was the same day patients in the treatment arm received convalescent plasma. Follow-up samples were taken approximately 1 week later (median 7 days, range 3–56 days). Healthy control pre-pandemic sera came from subjects enrolled in a prospective cohort study at the travel medicine and vaccination clinic of Erasmus MC (MEC-2014-398). Sera were heat-inactivated for 30 min at 56 °C. Bronchoalveolar lavage (BAL) samples were taken for routine clinical diagnostic purposes and leftover material was stored at below −20 °C until further analysis.

### Clinical Data

Clinical data, including age, sex, days of disease duration, vital parameters, medical history, and results of pathogen identification tests, were extracted from patient electronic medical files. Public registries were consulted for additional survival data. Three-day average Sequential Organ Failure Assessment (SOFA) scores and ratios of partial pressure of oxygen in arterial blood to fraction of inspired oxygen (P/F) were calculated based on the vital parameters available of the first 3 days of ICU admission at the time point closest to 6 a.m. Age and medical history were used to calculate a Charlson Comorbidity Index (CCI) for each ICU patient. All COVID-19 patients were diagnosed by a positive respiratory sample using a real-time quantitative PCR test (RT-qPCR) for SARS-CoV-2. For ICU patients with COVID-19, frequent measurements of RT-qPCR data (Ct values) were available. Clearance of viral RNA from the respiratory tract was defined as a single Ct value above 35, without a decrease in Ct value in subsequent samples. Time to clearance was calculated as the number of days between inclusion into the study and the day of viral clearance.

### Clinical Severity Grading

COVID-19 severity was graded using the 10-point clinical progression scale, published by the WHO Working Group on the Clinical Characterization and Management of COVID-19 infection, which classifies COVID-19 cases as mild, moderate, or severe, based on the level of supportive care the patient requires, with a score of 10 indicating fatal disease [14]. The highest score that was reached during the course of the disease was used to classify the patients. COVID-19 cases were classed as mild (WHO score 1–3 points), moderate (4–5 points), severe (6–9 points), and fatal (10 points).

### Serum Pools and Antibody Purification

Sera (*n* = 7) in which the highest concentration of IFN-α2 Abs was detected were pooled and a separate pool of negative sera was made as a control. To remove any serum factors that could interfere with the IFN blocking assay, pools were subjected to protein G column affinity chromatography to obtain purified Ig preparations, using a Proteus protein G antibody purification kit (Bio-Rad Laboratories, UK).

### rVSV ΔG GFP Rescue and Production

Replication-restricted recombinant Vesicular Stomatitis Virus encoding Green Fluorescent Protein (VSV ΔG GFP) was rescued as described previously [15]. Propagation to high titers was achieved by infecting HEK-293T cells transfected with VSV-G 24 h prior with a multiplicity of infection of 0.1 of rescued virus. Supernatant was collected after 48 and 72 h, cleared by centrifugation at 2000×*g* for 5 min and stored at −80°C. Titers were determined by preparing 10-fold serial dilutions in Opti-MEM I (1X) + GlutaMAX (Gibco). Aliquots of each dilution were added to monolayers of 2 × 10^4^ Vero cells in the same medium in a 96-well plate. Plates were incubated at 37°C overnight and then scanned using an Amersham™ Typhoon scanner. Infected cells were quantified using ImageQuant TL software (GE Healthcare Life Sciences). Vero cells were maintained in Dulbecco’s modified Eagle’s medium (DMEM, Gibco) supplemented with 10% F, 100 μg/mL streptomycin, 100 U/mL penicillin, 20mM Hepes (Lonza), and 1mM sodium pyruvate (Gibco). HEK-293T cells were maintained in DMEM supplemented with 10% FBS, 100 μg/mL streptomycin, 100 U/mL penicillin, 1X non-essential amino acids (Lonza), and 1mM sodium pyruvate (Gibco). All cell lines were maintained at 37 °C in a humidified CO_2_ incubator.

### IFN-α Neutralization Assay

A549 cells were maintained in F12 medium, supplemented with 10% FBS, 100 μg/mL streptomycin, and 100 U/mL penicillin. Cells were plated in 96-well cell culture plates and incubated at 37 °C for 24 h before infection. Purified Ab or serum was pre-incubated for 1 h at 37 °C with PEG-IFN-α2a (Roche Pharmaceuticals) at a concentration of 3 ng/mL, the minimum concentration causing >90% GFP+ plaque reduction in optimization experiments. Both serum and purified antibody from pooled positive (*N* = 7) samples caused strong functional inhibition of IFN-α2a but not of IFN-λ1 (supplementary figure 1).

Confluent monolayers of A549 cells were infected with 500 Plaque Forming Units (PFU) per well of replication-restricted recombinant Vesicular Stomatitis Virus encoding Green Fluorescent Protein (rVSV ΔG GFP) in culture medium containing 2% FBS, after at least 4 h of incubation with purified Ig or serum, both with and without PEG-IFN-α2a. Infections were performed under Biosafety Level II (BSLII) conditions. GFP positive plaques were quantified 8–24 h after infection using an Amersham™ Typhoon scanner with ImageQuant TL colony counting software (GE Healthcare Life Sciences). IFN neutralizing activity was determined by calculating the ratio of plaque neutralization in IFN+ serum/Ig incubated wells and IFN incubated wells, corrected for low-level VSV neutralization in some sera. All functional IFN blocking experiments were performed in triplicate. A control condition was included using IFN-λ1 (10 ng/mL) on A549 cells.

### Assay Validation

For detection of binding Abs against Human IFN-α2*,* a commercially available ELISA was used (BMS217 from Invitrogen) according to the manufacturers’ instructions. The captured antigen used for this kit was IFN-α2c. The first publication describing IFN-α2 Abs in COVID-19 patients showed that IFN-α2 binding sera reacted with all 13 subtypes of IFN-α [6]. A human anti-IFN-α2 standard included in the kit was used as a positive control. To determine the minimum cutoff for binding IFN-α2 Abs, pre-pandemic sera from healthy individuals were used as negative controls. The 99th percentile IFN-α2 Abs concentration found in healthy controls was 165 ng/mL, which was used as the cutoff to define IFN-α2 Abs positive sera. All sera above this cutoff were tested individually in IFN-α2 neutralization assay in 1:10 dilution and 300 pg/mL of IFN-α2, corresponding to 3 ng/mL in undiluted serum. Sera were considered neutralizing if they reduced IFN-α2 activity by at least 50%.

### SARS-CoV-2 Serology

Total SARS-CoV-2 Receptor Binding Domain–specific Ig was detected using the Wantai SARS-CoV-2 ELISA (Beijing Wantai Biological Pharmacy Enterprise, China) as previously described [16]. An in-house plaque reduction neutralization test (PRNT 50) was used to quantify virus-neutralizing Ab as previously described [17].

### Statistical Analysis

Data analysis was performed using SPSS statistics version 25 (IBM), Prism version 9 (GraphPad), and Excel 2016 (Microsoft). Comparisons of continuous variables between patient groups were performed using Student’s *t*-tests for normally distributed data (e.g., age, days of disease duration, and clinical scores) and the Mann-Whitney *U* tests for data following a skewed distribution (e.g., IFN-α2 Abs and SARS-CoV-2 ELISA results, PRNT 50 titers). IFN-α2 Ab concentrations determined by ELISA were 10 Log transformed. Geometric mean titers (GMT) were calculated from PRNT 50 titers. Comparisons of categorical data were performed using the chi-squared test. Dose-response curves were plotted using 4-parameter nonlinear regression, least-squares method, with top and bottom constrained to ≥ 0 and ≤ 100 respectively. Survival curves were compared using the logrank (Mantel-Cox) test. Correlation analysis was performed using Pearson’s correlation for parametric and Spearman for nonparametric variables.

## Results

### Subject Characteristics

Baseline characteristics of COVID-19 (*N* = 282) and non-COVID-19 ICU (*N* = 46) patients and healthy control subjects (*N* = 103) are listed in Table 1. Mild (*N* = 100) and moderate (*N* = 43) cases were subjects included in the CONCOVID clinical trial, as convalescent donors or hospitalized patients receiving either convalescent plasma or standard of care. Of the CONCOVID donors, 3 had missing data on disease severity. Of the moderate cases, 33/43 required supplemental oxygen. Most severe (*N* = 98) and fatal (*N* = 38) COVID-19 cases were from an observational cohort study of ICU patients, as were non-COVID-19 ICU patients (Table 1).

**Table 1:** Baseline characteristics and IFN-α Ab status of COVID-19 cohorts stratified by disease severity and non-COVID-19 controls

	Non-COVID-19 (*N* = 150)		COVID-19 (*N* = 282)			
	Healthy controls (*N*=103)	ICU patients (*N* = 47)	Mild (*N* = 100)	Moderate (*N* = 43)	Severe (*N* = 97)	Fatal (*N* = 38)
Age (mean years ± SD)	27 ± 11.8	61 ± 11.8	42 ± 12.9	58 ±13.9	61 ± 12.1	67 ± 10.7
Female (*N*)	76 (74%)	19 (41%)	9 (9%)	9 (21%)	27 (28%)	8 (21%)
Study						
ICU prospective cohort (*N* = 149)	-	47 (100%)	-	-	73 (75%)	29 (76%)
CONCOVID: donor (*N* = 118)	-	-	99 (99%)	15 (35%)	1 (1%)	-
CONCOVID: patient (*N* = 61)	-	-	1 (1%)	28 (65%)	23 (24%)	9 (24%)
Healthy cohort (*N* = 103)	103 (100%)	-	-	-	-	-
Disease duration (mean days ± SD)	-	-	50 ± 14	28 ± 23.6	14 ± 7.3	14 ± 8.5
SARS-CoV-2 IgG (median ratio ± IQR)	-	-	14.49 ± 1.66	18.4 ± 7.60	18.4 ± 8.49	15.16 ± 17.75
SARS-CoV-2 PRNT 50 (GMT ± SD)	-	-	117 ± 4	184 ± 5	134 ± 7	47 ± 7
Anti-IFN-α2 positive (*N*)	1 (1%)	0 (0%)	4 (4%)	0 (0%)	7 (7%)	5 (13%)
Anti-IFN-α2 neutralizing (*N*)	0 (0%)	0 (0%)	1 (1%)	0 (0%)	7 (7%)	5 (13%)

*SD* standard deviation

Non-COVID-19 infections included viral, bacterial, and fungal pathogens (Supplementary table 1) with 11/47 (23%) being viral. These patients tended to have more comorbidities compared to COVID-19 ICU patients and more likely to suffer from multi-organ failure. Both COVID-19 and non-COVID-19 ICU patients suffered from similar levels of respiratory insufficiency (Supplementary table 1).

In the COVID-19 group, mean age increased with disease severity, which was statistically significant in a one-way ANOVA comparing the 4 severity classes (*F* = 56, *P* < 0.0001). COVID-19 patients were more likely to be men compared to non-COVID-19 ICU patients and healthy controls. Almost all (99%) of mild cases were convalescent plasma donors, of whom 91% were men (women were initially excluded from plasma donation). Mild and moderate cases were sampled longer after onset of COVID-19 symptoms (28 and 50 days), compared to those with severe or fatal COVID-19, who were more often sampled in the acute phase of the disease (mean 14 days disease duration). SARS-CoV-2 binding and neutralizing antibody levels were lower on average in the mild and fatal groups, but this was not statistically significant in one-way ANOVA (SARS-CoV-2 Ig: *F* = 2.2, *P* = 0.09, PRNT GMT: *F* = 2.1, *P* = 0.11).

### IFN-α2 Abs

**Fig. 1:**
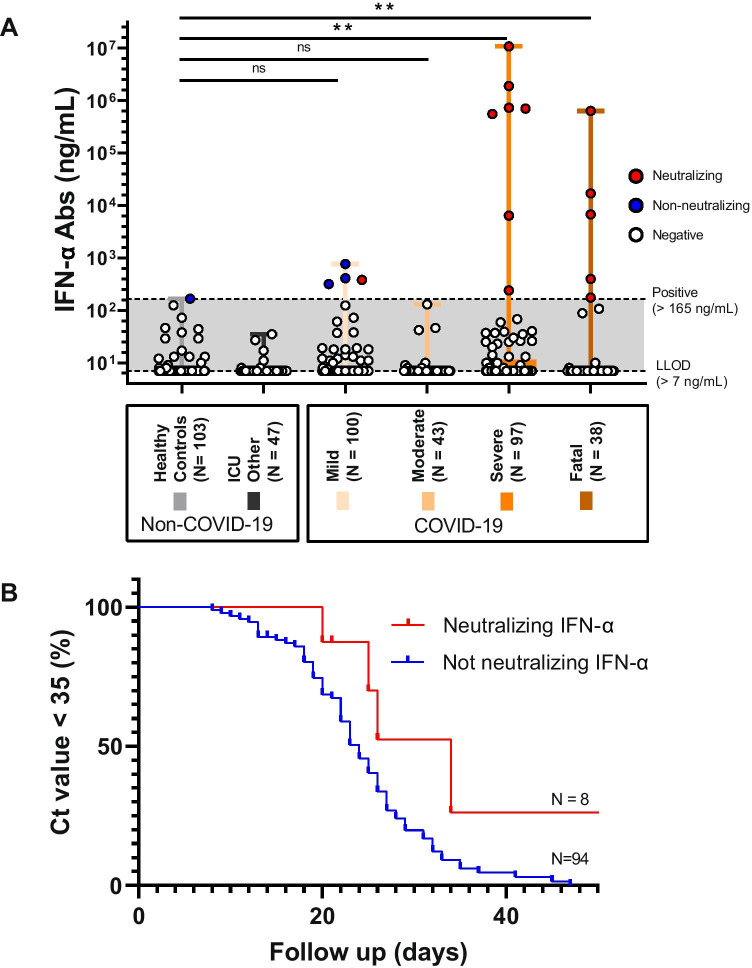
Quantitative levels and functional properties of IFN-α Abs in COVID-19 patients. Plot of IFN-α Abs concentrations in COVID-19 patients, healthy controls and non-COVID-19 patients. Dots are individual values (A). (B) Survival curve plotting time to viral clearance in COVID-19 ICU patients with neutralizing IFN-α Abs (red line, *N*=8) versus no neutralizing IFN-α Abs (blue line, *N*=94). ns = not significant, ** = *P* <0.01

Overall, 16 out of 282 COVID-19 patients (6%) tested in this study had ELISA-detectable IFN-α2 Abs, with proportions increasing with disease severity (Fig. 1A, Table 1). Of these, 13 were found to neutralize IFN-α2 in our rVSV-based assay, 3/4 positive sera from mild COVID-19 subjects being non-neutralizing. Contrary to the 3 non-neutralizing sera from mild cases, the serum that did neutralize IFN-α2 was taken in the acute stage of the disease (15 days after symptom onset). No healthy controls or non-COVID-19 ICU patients had neutralizing IFN-α2 Abs.

Bronchoalveolar lavage (BAL) samples were available from 21 COVID-19 ICU patients, 3 of whom had detectable serum IFN-α2 Abs (2 neutralizing, 1 non-neutralizing). The patient with neutralizing serum IFN-α2 Abs (6.38 × 10^5^ ng/mL) also had ELISA-detectable IFN-α2 Abs in their BAL sample (201 ng/mL), whereas the rest tested below the cutoff defined for serum.

To determine if IFN-α2 Abs could appear later in the course of the disease, patients who had initially tested negative and who had follow-up sera available (*N* = 33) were followed up weekly until discharge or death. Of these patients, one developed IFN-α2 Abs, with increasing levels until they exceeded the cutoff value at 3 weeks after the initial sample was taken (supplementary figure 2). However, none of the sera taken from this patient neutralized IFN-α2.

### Risk Factors for Neutralizing IFN-α2 Abs and Effect on Clinical and Virological Outcomes

All COVID-19 cohorts combined (*n* = 282) were analyzed to identify factors associated with having neutralizing IFN-α2 Abs. Mean age of COVID-19 patients with neutralizing serum IFN-α2 Abs was significantly higher compared to patients with negative or non-neutralizing IFN-α2 Abs (Table 2). The proportion of women in the group with neutralizing IFN-α2 Abs was also significantly higher. Excluding convalescent plasma donors, who were selected based on male sex and mild to moderate disease, lead to substantial reduction in this difference between men and women, which was no longer statistically significant (6 vs 13%, *P* = 0.11)

**Table 2:** Comparisons between IFN-α Abs positive and negative COVID-19 patients

	IFN-α neutralizing (*N* = 13)	IFN-α Abs negative (*N* = 269)	*P*
Age (mean years ± SD)	66 ± 9	53 ± 17	<0.0001
Female (*N*)	6 (46%)	47 (17%)	0.043
Disease duration (mean days ± SD)	11 (± 5)	31 (± 22)	<0.0001
SARS-CoV-2 IgG (median ratio ± IQR)	16.72 (14.94)	14.79 (11.78)	0.411
SARS-CoV-2 PRNT 50 (GMT ± SD)	403 (± 8)	122 (± 5)	0.190

*PRNT 50* 50% plaque reduction neutralization titer, *GMT* geometric mean titer

No difference in 60-day survival was found when comparing critical and severe COVID-19 patients who had neutralizing IFN-α2 Abs (*n* = 12) versus those who did not have (*n* = 150) neutralizing levels in univariate analysis (HR 1.90, 95% CI 0.56–6.40). Among COVID-19 patients for which daily viral RNA measurements were available, those with neutralizing IFN-α2 Abs had a longer time to viral clearance from the respiratory tract patients without (median 24 days vs 34 days, HR 2.3, 95% CI 1.2–4.4, Fig. 1C).

### IFN-α Abs in Convalescent Plasma Therapy

Of 118 COVID-19 convalescent plasma donors screened, 3 (3%) had detectable serum IFN-α2 Abs, but none was neutralizing in our rVSV assay. Plasma from these 3 ELISA positive donors had not been used to treat COVID-19 patients. Of the hospitalized COVID-19 patients who received either convalescent plasma or placebo as part of the CONCOVID clinical trial (*N* = 61), 4 (7%) had detectable serum IFN-α2 Abs, all of which were neutralizing. Of the trial subjects that were IFN-α2 Abs negative before treatment, no IFN-α2 Abs appeared in the sera taken after treatment in either the intervention or placebo arm.

## Discussion

In this study, we tested the presence of IFN-α Abs in COVID-19 patients and determined their relevance for convalescent plasma treatment and relation to disease outcomes. Using a commercially available ELISA, we detected IFN-α Abs at a similar rate as previously described in patients with life-threatening COVID-19, which decreases in moderate and mild cases [6]. Severe COVID-19 is associated with a state of immune hyper activation and the production of a broad spectrum of autoreactive antibodies, which in turn have not been linked to clinically apparent auto-immune disease [18, 19]. This underlines the need for testing the functional properties of auto-antibodies found and relating them to clinical phenotype. In this study, we found associations between neutralizing IFN-α Abs and COVID-19 disease severity, age, and time to clearance of viral RNA from the respiratory tract. An association between IFN-α Abs and mortality did not reach statistical significance in 60-day survival analysis, although fatal COVID-19 cases had the highest proportion of positive anti-IFN-α sera. Recently, an analysis of a cohort of ICU admitted COVID-19 patients was published, which correlates neutralizing type I IFN-Abs with a higher mortality rate from COVID-19 induced multi-organ failure [20]. We found no clear association between sex and the risk of IFN-α2 Abs. In contrast, Bastard et al. showed a strong overrepresentation of men among COVID-19 patients with type I IFN-Abs, and an X-linked genetic association was proposed [6]. However, since male sex is also associated with a higher severe COVID-19 and subsequent in-hospital mortality [21], there is a possibility that the sex association is mediated by disease severity. Due to selection bias introduced by the (overwhelmingly male) CONCOVID donors, we excluded these from the analysis comparing IFN-α2 Abs between the sexes. The resultant analysis included mostly severe and fatal COVID-19 patients and lacked sufficient statistical power to detect any difference between the sexes. Disease severity could also mediate the correlation between IFN-α Abs and age, although a more recent study by Bastard et al. showed that in a healthy population, the prevalence of IFN-Abs also rises with age, especially in over 70s [22].

Our finding that most patients who had detectable IFN-α Abs already tested positive at the earliest available time point, and the appearance of IFN-α Abs later during the course of the disease was rare, suggests that these individuals already harbored anti-IFN B cell clones before they were infected. The SARS-CoV-2 infection may have boosted the anti-IFN-Abs rather than being causally linked to the induction of auto-immunity. Patients in whom IFN-Abs are found mostly do not have any previous history of frequent severe infections in their medical history, viral or otherwise, which raises the question whether immune deficiency caused by IFN-Abs is specific for SARS-CoV-2 or primary viral infections in general. Outside of a pandemic with a newly emerged pathogen, the great majority of infections occurring in later adulthood are secondary, where any innate immune defect caused by IFN-Abs could be masked by the benefits from adaptive immunity acquired earlier in life, before anti-IFN auto-immunity had developed. Alternatively, there could be a lack of awareness among clinicians regarding the existence of such immune deficiencies, leading to under diagnosis. Vaccination with replication competent live-attenuated vaccines represents another form of primary viral infection and a recent case series of yellow fever vaccine–associated viscerotropic and neurotropic diseases (YEL-AND/AVD) identified serum IFN-Abs in 3 individuals who received the vaccine at 57, 59, and 62 years of age [23].

Although several sera from COVID-19 convalescent plasma donors had ELISA-detectable IFN-α2 Abs, they did not neutralize the same levels of IFN-α2 compared to acute COVID-19 patients and their plasma had not been used for convalescent plasma treatment. Even when their plasma had been used, this is not expected to result in neutralizing IFN-α2 Abs levels in the recipient, considering a single transfusion of 300 mL plasma would be diluted in the total plasma volume of the patient. In the CONCOVID trial, plasma recipients were more likely to have IFN-α2 Abs compared to plasma donors.

Sensitivity and specificity of IFN neutralization assays are influenced by the concentration of IFN used. In our neutralization assay, we used 1/10 diluted serum and an IFN-α concentration of 300 pg/mL, which corresponds to a neutralizing capacity of 3 ng/mL of IFN-α in circulating blood. This concentration was optimized for use in our assay based on VSV infection of A549 cells, and is significantly higher compared to IFN-α2 plasma levels commonly observed in plasma COVID-19 during the acute phase of disease [4]. In their original publication, Bastard et al. used an IFN-α concentration of 10 ng/mL for 1/10 diluted plasma, which they recently repeated in an assay with a lower IFN-α concentration of 100 pg/mL (corresponding to 1 ng/mL in circulating blood), resulting in a detection rate of neutralizing IFN-α Abs of 13.6% in critically ill patients and 6.8% of severely ill COVID-19 cases [22]. This rate closely resembles our findings in COVID-19 patients with similar disease severity.

This study only examined auto-antibodies against IFN-α2, which is the most common subtype that COVID-19 patients have antibodies against [6]. In the study by Bastard et al., patients with neutralizing IFN-α2 abs were found to also neutralize other IFN-α subtypes; however, it is not known whether this applies to the cases in this study. We also did not test for abs against IFN ω, which were found in 1.3% of life-threatening COVID-19 cases without IFN-α Abs by Bastard et al. [6]. The omission of this IFN from our analysis is one of the limitations of our study. In conclusion, we confirm previous finding that IFN-α2 Abs can be detected in COVID-19 patients, with neutralizing levels being most common in critically ill COVID-19 patients in the acute stage of the disease. In these patients, neutralizing IFN-α2 Abs were associated with delayed viral clearance from the respiratory tract. In contrast, we did not find neutralizing IFN-α2 Abs in critically ill ICU patients with respiratory illness caused by other infectious diseases, or in COVID-19 convalescent plasma donors. We did not detect any cases where convalescent plasma transfusion was associated with the appearance of IFN-α2 Abs in the recipient.

## Supplementary Information

Below is the link to the electronic supplementary material.

Supplementary file1 (DOCX 193 kb)

## Data Availability

All data presented in the current manuscript is available on request to the corresponding author.
