# Supplementary material for: Interferon-α2 Auto-antibodies in Convalescent Plasma Therapy for COVID-19
Source: J Clin Immunol. 2021 Nov 12;42(2):232–9. doi: 10.1007/s10875-021-01168-3 (PMC8586830; doi:10.1007/s10875-021-01168-3)
Supplement: Supplementary file 1 — Supplementary file1 (DOCX 193 kb) [file 10875_2021_1168_MOESM1_ESM.docx]

# Supplementary material

Interferon-α auto-antibodies in convalescent plasma therapy for COVID-19

Matthijs P. Raadsen,1 Arvind Gharbharan,2 Carlijn.C.E. Jordans,2 Anna Z. Mykytyn,1 Mart M. Lamers,1 Petra B. van den Doel,1 Henrik Endeman,3 Johannes P.C. van den Akker,3 Corine H. GeurtsvanKessel,1 Marion P.G. Koopmans,1 Casper Rokx,2 Marco Goeijenbier,13 Eric C.M. van Gorp,1 Bart J.A. Rijnders,2 Bart L. Haagmans,1*

1Viroscience department, Erasmus MC, Rotterdam, The Netherlands.

2Department of Medical Microbiology and Infectious diseases, Erasmus MC, Rotterdam, The Netherlands. .

3Intensive Care department, Erasmus MC, Rotterdam, The Netherlands.

Correspondence: Bart L. Haagmans, Viroscience department, Erasmus MC, Rotterdam, The Netherlands (b.haagmans@erasmusmc.nl).

# Tables

Table S1: Pathogens identified in critical infectious respiratory disease in the ICU.

|  | COVID-19  N= 102 | Non-COVID-19  N = 47 | p value |
| --- | --- | --- | --- |
| Charlson comorbidity index (Mean ± SD) | 2.7 ± 1.7 | 4.2 SD ± 2.1 | <0.0001 |
| SOFA score (Mean ± SD) | 6.4 ± 3.0 | 8.3 ± 3.5 | 0.001 |
| P/F ratio (Mean ± SD) | 209 ± 84 | 236 ± 112 | 0.103 |
| Primary diagnosis (N) |  |  |  |
| Aspiration pneumonia |  | 9 |  |
| COPD Exacerbation |  | 2 |  |
| HAP | 4 | 3 |  |
| Neutropenic Sepsis |  | 1 |  |
| CAP | 98 | 18 |  |
| Pneumonia following chest injury |  | 1 |  |
| Sepsis |  | 11 |  |
| Meningitis |  | 1 |  |
| Soft tissue infection |  | 1 |  |
| Pathogen Detected (N) |  |  |  |
| SARS-CoV-2 | 102 |  |  |
| Influenza virus |  | 5 |  |
| Respiratory Syncytial Virus |  | 3 |  |
| Rhinovirus |  | 2 |  |
| Seasonal CoV |  | 1 |  |
| S. Pneumoniae |  | 8 |  |
| E. Coli |  | 6 |  |
| S. Aureus |  | 4 |  |
| Klebsiella Spp |  | 5 |  |
| Pseudomonas Spp |  | 3 |  |
| Citrobacter Spp |  | 2 |  |
| Corynebacterium Spp. |  | 1 |  |
| Enterococcus Spp |  | 4 |  |
| Serratia Spp |  | 2 |  |
| Streptococcus Spp |  | 3 |  |
| H. Influenzae |  | 1 |  |
| Enterobacter Spp |  | 1 |  |
| Neisseria Spp |  | 1 |  |
| Mycoplasma Spp |  | 1 |  |
| Proteus Spp |  | 1 |  |
| Aspergillus Spp |  | 5 |  |
| Pneumocystis Jiroveci |  | 1 |  |
| Autoimmune pneumonitis |  | 1 |  |
| Unknown |  | 7 |  |

*SD = Standard Deviation, COPD = Chronic Obstructive Pulmonary Disease, HAP = Hospital-acquired pneumonia, CAP = Community-acquired pneumonia, OHCA = Out-of Hospital Cardiac Arrest, SOFA = Sequential Organ Failure Assessment, P/F* *= Ratio of partial pressure of oxygen in arterial blood to fraction of inspired oxygen.*

# Figures


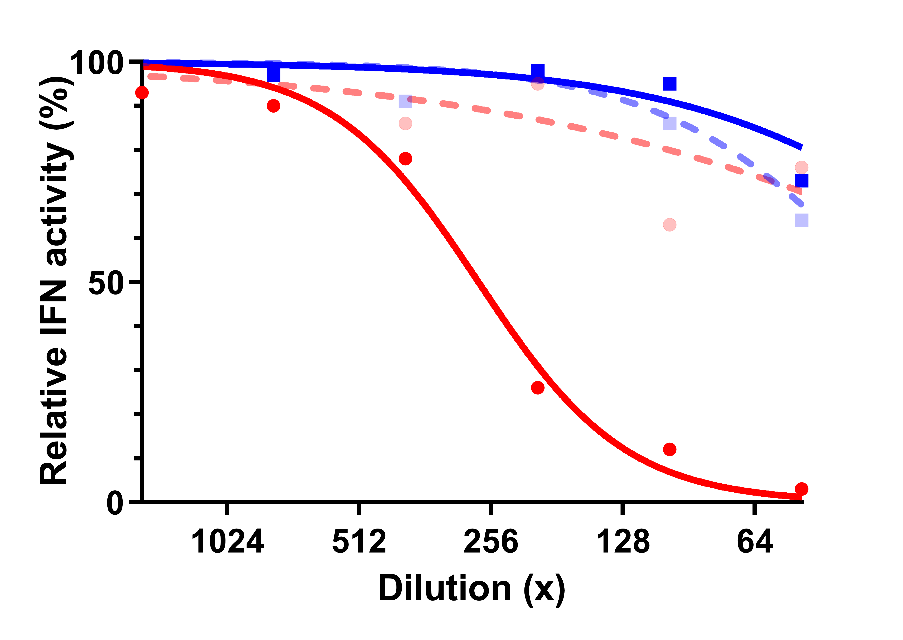


Figure S1: Sigmoidal curve showing inhibition of IFN activity by IFN-α Abs positive (red) and negative (blue) pools on A549 cells. Solid lines/symbols represent PEG IFN-α activity, transparent lines/symbols represent IFN-λ1 activity.


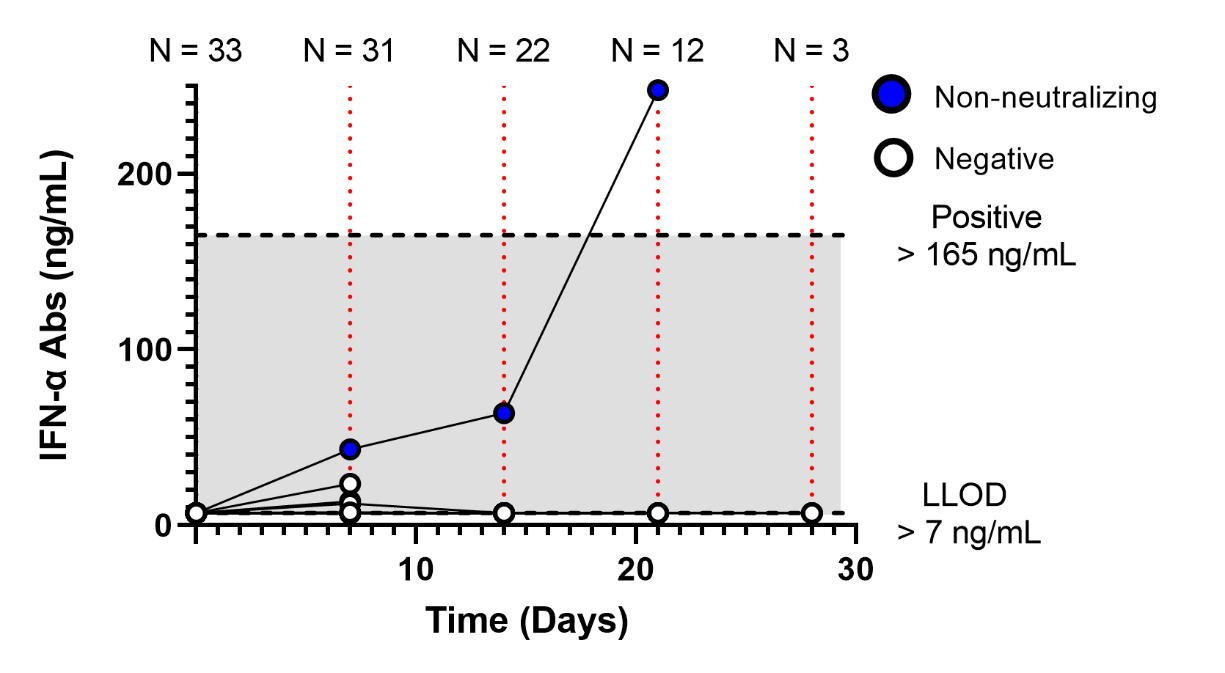


Figure S2: Longitudinal individual IFN-α Ab concentrations in COVID-19 ICU patients who tested negative at baseline.
